# Removing of cationic dyes using self-cleaning membranes-based PVC/nano-cellulose combined with titanium aluminate

**DOI:** 10.1007/s11356-023-27691-x

**Published:** 2023-06-07

**Authors:** Aya Abd El Aziz Elfiky, Mahmoud F. Mubarak, Mohamed Keshawy, Ibrahim El Tantawy El Sayed, Thanaa Abdel Moghny

**Affiliations:** 1 Petrolum Applications Department, Egyptian Petroleum Research Institute (EPRI), Ahmed El-Zomer, Nasr City, Cairo Egypt; 2 Department of Chemistry, Faculty of Science, Menoufia University, 32511, Shebin El Koom, Menoufia Egypt

**Keywords:** Self-cleaning, Nanocomposite, Membrane, Nanofiltration, Hydrophobicity, Cationic dye

## Abstract

This research used the phase inversion approach to construct polyvinyl chloride nanocellulose@titanium aluminate nanocomposite membranes (PVC/NC@TALCM) to adsorb and filter dye from wastewater. FTIR, XRD, and SEM were used to determine the adsorptive nanocomposite membrane that had been synthesized. The thermal and electrical properties measurements were carried out using a static system. The influence of several adsorbent dosages, pH, and dye concentrations on the nanocomposite membrane’s adsorption ability was investigated. Using a dead-end filtration system, the PVC-NC@TALCM was evaluated as a pressure filtration membrane system. It was found that 98.6% of MB dye was removed by PVC-NC@TALCM membrane, which was loaded with 5% titanium aluminate at pH 10. The kinetic adsorption studies indicated that the adsorption of MB onto the PVC-NC@TALCM nanocomposite membrane obeys pseudo-second-order that indicates the chemosorption process. The isotherm data were described using Freundlich and Langmuir models, and the Freundlich isotherms were shown to be more closely match the experimental data than the Langmuir model. Finally, the PVC-NC@TALCM nanocomposite membrane was economical, environmentally friendly, and self-cleaning.

## Introduction

Wastewater is a major concern as it contains pollutants that harm the environment and human health around the world. High amounts of dyes, heavy metals that discharged into water bodies, create substantial health and environmental problems, as well as a rise in wastewater treatment costs. Thereby, the removing of dyes and heavy metals from drinking and wastewater is essential for environmental and human health protection (Sunil et al. 2021).

Researchers have used standard techniques for treating dye solutions, such as ultrafiltration membrane, ion exchange, electrocoagulation, advanced oxidation, photocatalytic degradation, coagulation and flocculation, and phytoremediation (Teng et al. 2022). However, the abovementioned methods are unsuccessful due to the processing of large quantities of sludge, harmful by-products, bio-resistant organisms, and complex operational procedures (Oladoye et al. 2022). Due to its low cost, membrane nanofiltration has long been a promising technique in industrial wastewater treatment areas (Gidstedt et al. 2022).

The membrane filtration method suffers from concentration polarization and membrane fouling, leading to a decrease in separation efficiency, an increase in operating costs, and a shortening of membrane lifetime. Polymeric membranes are extensively employed in water purification and wastewater treatment (Pan et al. 2022).

Membrane types such as microfiltration, nanofiltration, ultrafiltration, catalytic, and conducting membranes have been developed for wastewater treatment and bio-processing (Wang et al. 2021). This paper created a one-step impregnation of nanomaterials such as nanocellulose on membrane surfaces to provide self-cleaning properties when exposed to polluted wastewater (Wang et al. 2022a, b, c).

Polyvinyl chloride membranes (PVC) are widely used due to their hardness, resistance to abrasion, acid, alkali, and microbial corrosion, but their hydrophobicity can cause fouling when processed with protein-like substances (Pankiew et al. 2021). In addition, PVC has gained research attention as a separation membrane due to its low biofouling propensity. Self-cleaning membranes provide a solution to the challenge of keeping membranes permeable, clean, and functional (Nthunya et al. 2022).

Cellulose is the most prevalent polymeric raw material derived from the biosphere. It is composed of crystalline cellulose, hemicellulose, lignin, mineral, wax, and ash. Nanocellulose (NC) can be used as an adsorbent material for water treatment, attaching pollutants such as heavy metal ions and dyes (Perumal et al. 2022).

The composite technique of impregnating porous substrates with multiple NC grades has sparked renewed interest in producing adsorbents (Chen et al. 2022). Nanometal oxides such as TiO_2_ nanoparticles are potent inorganic materials, boosting permeability, surface hydrophilicity, mineralization, and self-cleaning/antifouling capabilities (Voisin et al. 2021).

This research aims to prepare a new nanocomposite membrane filter based on PVC/nano cellulose and titanium aluminate. Such membrane will be evaluated as an adsorbent to remove MB dye at different concentrations. The Effect of the TiAl_2_O_4_ concentration on the physical and chemical properties of the membranes will be studied. Parameters such as dye concentration, pH, membrane dose, and temperature will be studied. Various isothermal and kinetic models will be applied to verify the adsorption behavior and desorption process. Experimental results of dye batch adsorption will be investigated using PVC-NC @ TALCM membrane.

## Experimental

### Materials

The agricultural wastes were received from Egyptian farms as the source of cellulose. Polyvinyl chloride (PVC), tetrahydrofuran (THF), dimethyl formamide (DMF), sodium hydroxide (NaOH), titanium acetate Ti(CH_3_COO)_2_.2H_2_O, ammonium hydroxide NH_4_OH, titanium chloride (TiCl_4_), and methylene blue (MB) from Sigma Aldrich were utilized throughout the investigation. Distilled water from the central lab was also employed. Other chemical products were used, such as distilled water, HCl, isopropyl alcohol, and NH_4_OH (25%) solutions. In this work, all of the chemicals were used under analytical grade.

### Purification of agricultural wastes

Agricultural wastes with varying cellulose concentrations, such as rice straw, were collected and dried in the sun before being chopped into little pieces (1–3 mm) (Debnath et al. 2021). The cut wastes were washed with distilled water and acetone, then dried overnight in an oven at 60 °C and chosen for other cellulose. Ten grams of the cellulosic materials undergo to alkali treatment with 250 ml sodium hydroxide (1% wt/v) solution for 5 h and refluxed at 120 °C to remove the hemicelluloses. The insoluble residue (cellulose) was collected by filtering at the end of the extraction and rinsed extensively with distilled water until the filtrate was neutral. The resultant cellulosic materials were dried in the air before being mixed with dimethylsulfoxide (DMSO) and heated in a water bath at 80 °C for 3 h. The product was then filtered, washed with distilled water, and air-dried (Shaheen and Emam 2018).

### Preparation of nanocellulose

**Fig. 1:**
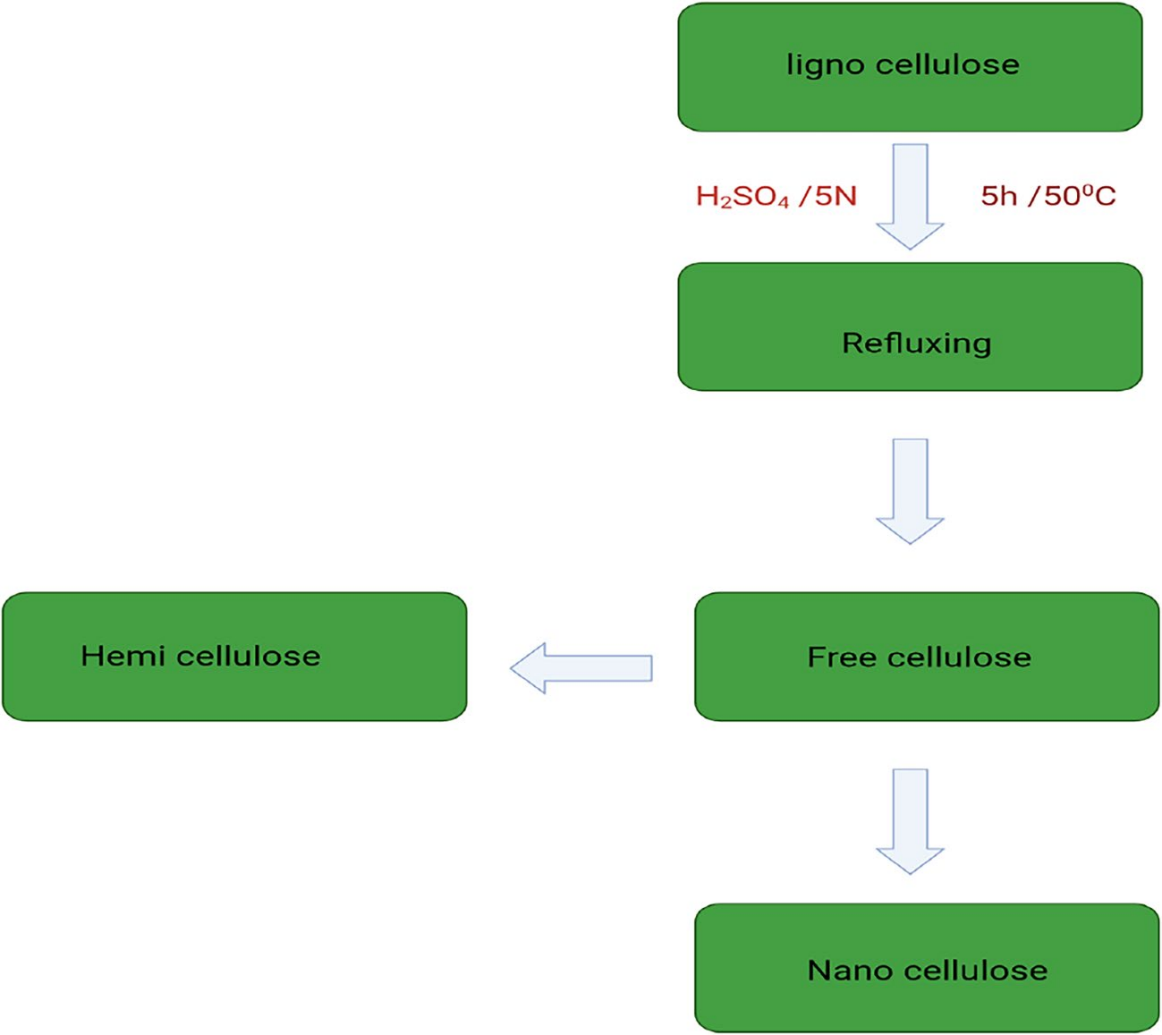
Preparation of nanocellulose

Nanocellulose (NC) was synthesized in spherical form rod-like highly crystalline nanocrystals. In this respect, 5 g of nanocellulose was obtained from 15 g of lignocellulosic material by acid hydrolysis and refluxing of dignified and hemicellulose (free cellulose) with 250 ml of 5 N sulfuric acid for 5 h at 50 °C under vigorous agitation to remove amorphous area and form cellulose nanocrystals as shown in Fig. 1. The hydrolysis was stopped by adding fivefold the quantity of water to the reaction mixture. Before centrifugation, the resulting mixture was allowed to cool to room temperature. The fractions were cleaned with distilled water and centrifuged regularly. After at least five washing cycles, centrifugation ended, and the supernatant liquor became turbid (Vincent and Kandasubramanian 2021).

### Preparation of titanium dioxide nanoparticles via sol–gel method

**Fig. 2:**
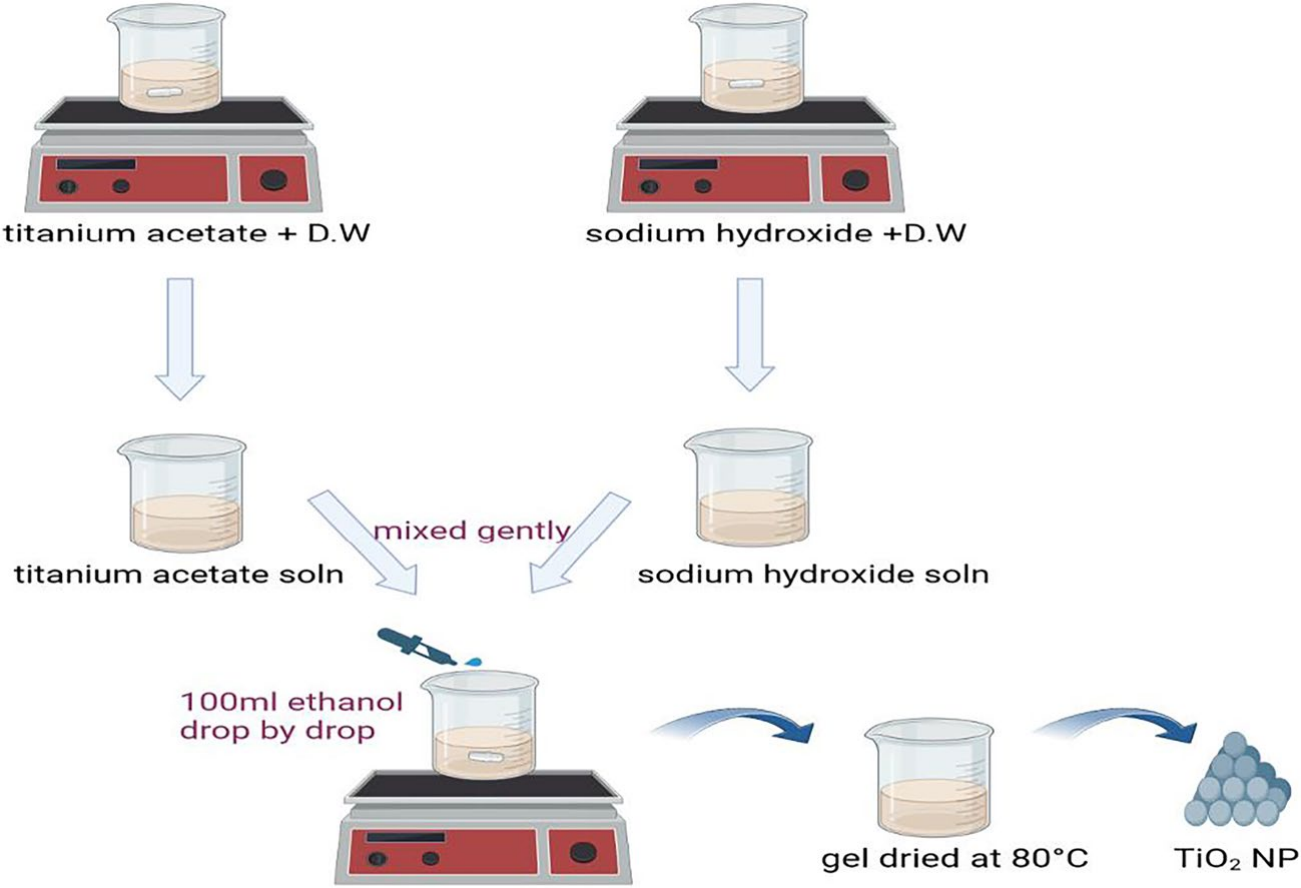
Titanium dioxide nanoparticle preparation

Briefly, to prepare a titanium acetate solution, 20 g Ti (CH_3_COO)_2_ 2H_2_O was combined with 150 ml distilled water and agitated for 20 min at 35 °C. To prepare the NaOH solution, 80 g (1% w/v) NaOH powders were dissolved in 80 ml water and stirred for 20 min at 35 °C. Both resolutions were mixed gently, and the titration reaction went smoothly, with 100 ml ethanol added drop-by-drop and vigorous stirring for 90 min until a gel-like result was obtained. To create TiO_2_ nanoparticles, the gel was dried at 80 °C overnight and calcinated at 250 °C in the furnace for 4 h (Tesfaye Jule et al. 2021). Figure 2 depicts the production of TiO_2_ nanoparticles using NaOH and is expressed by the following Eq. 1.

1 $${\mathrm{Ti}\left({\mathrm{CH}}_{3}\mathrm{COO}\right)}_{2}.2{\mathrm{H}}_{2}\mathrm{O}+2\mathrm{NaOH}\to {\mathrm{TiO}}_{2}+2{\mathrm{NaCH}}_{3}\mathrm{COO}+{\mathrm{H}}_{2}\mathrm{O}$$

### Synthesis of titanium aluminate nanocomposite

Although metallic alkoxides have high reactivity with water, which favors the hydrolysis process and results in the immediate precipitation of the metallic hydroxide, the alkoxides were employed as precursors in the synthesis of titanium aluminate in our work. In this regard, 43.92 g of titanium sec-butoxide and 63.57 g of aluminum sec-butoxide were transferred to a 1-L three-necked glass flask. After that, in the glove box, these alkoxides were shaken well under a nitrogenous atmosphere in the three-ended balloon with a Teflon blade coated with thin-film metallic glass (TFMG) for 30 min to be homogenized. The alkoxides moisture will become almost viscous and exhibit yellow transparent color. To solve the alkoxides moisture, add 100 ml isopropyl alcohol and shake for another 2 h. Finally, 30 ml of 1.7 M H_2_O and 0.5 M HCI and solution in isopropyl alcohol solution were added under constant shaking to avoid the inactivity atmosphere of the balloon (Ha et al. 2020).

### Composite membranes fabrication

**Fig. 3:**
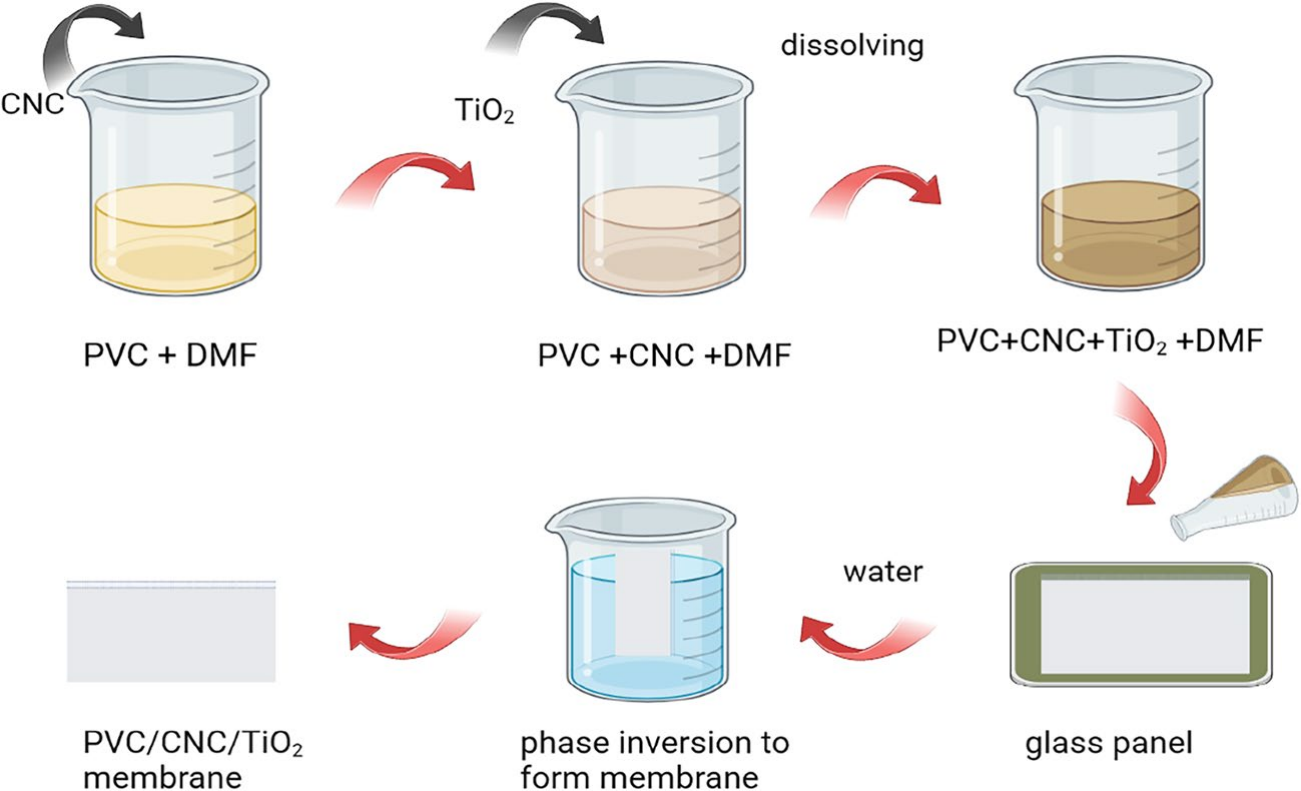
Preparation of nanocomposite membrane

The asymmetric membranes were made using the phase inversion approach as the following: the polymer solution, which included polyvinyl chloride (PVC) and nanocellulose (NC), was dissolved in dimethyl formamide (DMF), and the mixture was then mixed with titanium oxide nanoparticles and rapidly agitated for 24 h at 60 °C, as shown in Table 1, before being manually cast with a 150-mm-thick casting knife at room temperature on a clean glass plate. As illustrated in Fig. 3, for free-convective solvent evaporation, the membrane surface was exposed to air at ambient temperature (about 26 °C. After 60 s delay, a distilled water bath submerged the membranes at 26 °C. After 15 min, the polymeric skin layer was removed and immersed in distilled water for 24 h to remove all residual solvents (Deng and Li 2021). Digital caliper equipment (Electronic outside Micrometer, IP54 type OLR) was used to measure the thickness of the final membrane between 135 and 150 mm. Two processes must be completed to obtain the final membrane: first, the contact angle concentration must be optimized to get a membrane with the highest rejection and flux. The second stage examines the influence of different nanoparticle adsorbent concentrations on dye rejection (Sun et al. 2021).

**Table 1:** The composition of the membrane casting solution

Membrane sample no	PVC (%w/w)	NC (%w/w)	TiAl_2_O_4_(%w/w)	(wt%) DMF
1	15	0	0	100
2	15	0.15	0	100
3	15	0.15	0.15	100
4	15	0.15	0.45	100
5	15	0.15	0.75	100

### Membrane characterization

FTIR spectral analysis of membrane was performed using FTIR, model (Thermo Scientific Nicolet iS10 Spectrometer) produced by Thermo Fisher Scientific (USA) company in the spectral range from 4000 to 400 cm^−1^, the beam splitter is KBr/Ge mid-infrared optimized, with resolution 4 cm^−1^ (Park et al. 2021). X-ray diffraction (XRD) was employed to obtain the phases of the samples. X-ray diffraction (Philips PW3040/60 X, pert PRO P Analytical-Netherlands) was used to obtain the patterns. Continuous measurements were taken from 5 to 90° while the samples were on a glass slide. (Jain et al. 2022). All samples were observed by scanning electron microscope (SEM) model (Zeiss evo 10) attached with energy dispersive X-ray analysis (EDX) unit, with accelerating voltage 500 kV magnification14 × up to 100,000 × (Agarwalla and Mohanty 2022). TGA analysis was performed on the Perkin-Elmer TGA7 thermo balance. The dried samples were analyzed under a nitrogen atmosphere in the temperature range of 5–600 °C at a scanning rate of 20 °C/min.

### Dye adsorption experiments

At room temperature, 0.1 g of a PVC-NC@TALCM membrane was submerged in 50 mL of MB solution and swirled at 400 rpm. The free MB was analyzed at a wavelength of 664 nm and at different time intervals using UV–a visible spectrophotometer Shimadzu (UV-2600).

The influence of changing the initial dye concentrations (10, 15, 20, 25, 30 ppm), adsorbent dose (0.1, 0.5, 0.8 g), and pH (5, 7, 9) were studied. From Eqs. 2 and 3, both dye uptake efficiency (*R*%) and the adsorption capacity (*q*_e_) can be calculated.

2 $${\mathrm{q}}_{\mathrm{e}}=\frac{\left({\mathrm{c}}_{^\circ }-{\mathrm{c}}_{\mathrm{e}}\right)\mathrm{v}}{\mathrm{w}}$$

3 $$\mathrm{R}\left(\mathrm{\%}\right)=\frac{\left({\mathrm{c}}_{^\circ }-{\mathrm{c}}_{\mathrm{e}}\right)}{{\mathrm{c}}_{^\circ }}\times 100$$

where *W* is the membrane’s weight (g), *V* is the volume (L), and the equilibrium and starting concentrations of MB dye are *C*_e_ (mg/L) and *C*_o_, respectively [23].

### Self-cleaning assessment property of PVC/NC@TiAl2O4 membrane through the contact angle measurement

Cut the PVC/NC@TiAl_2_O_4_ membrane to the desired size and clean it thoroughly. Measure the contact angle using a contact angle goniometer and software. Repeat the measurement several times to ensure consistency. Calculate the mean and standard deviation of the contact angle measurements and compare them to a control sample to determine if the membrane exhibits a self-cleaning property. A surface with a low contact angle (less than 90°) indicates that the surface is hydrophilic and likely to exhibit self-cleaning behavior (Ismail et al. 2022).

## Result and discussion

### FTIR analysis of nanocomposite membranes

**Fig. 4:**
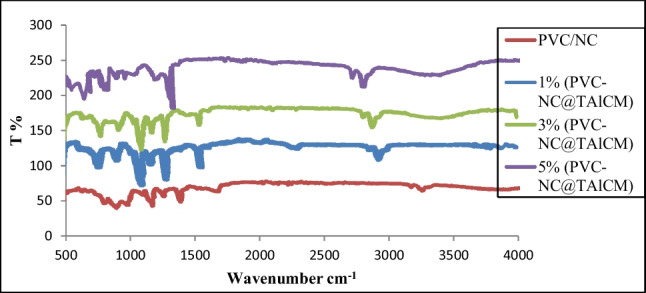
FTIR of PVC/NC and PVC-NC@TALCM with different ratios (1, 3, 5%)

The FTIR spectrum of PVC-NC and PVC-NC@TALCM membranes at different titanium aluminate concentrations is given in Fig. 4. Such spectrum exhibited bands at 2239 and 1331 cm^−1^, that may be assign to CH_2_’s asymmetric stretching and deformation vibrations, respectively, and the absorption peaks appeared at 1031 and 829 cm^−1^, may cause by CH’s in-plane and out-plane bending modes, are the distinctive PVC’s peaks. Whereas, the stretching vibration peak appeared at 3409 cm^−1^ may be the (OH) group of the nanocellulose. The two absorption peaks at around 1688 and 1401 cm^−1^ may be ascribed to the absorption of the bending vibrations of (C-O), as can be observed from the spectrum of PVC /NC) (Rana et al. 2021).

It is found that upon combining PVC/NC with TiAL_2_O_4_, the intensity of expansion − OH band increases from lower wavelengths 485, 486.4, and 479 cm^−1^, and moves to a higher wavelength 683, 633.8, and 544.99 cm^−1^, with increasing TiAL_2_O_4_ ratio from 1, 3, 5%, respectively. This may be due to the formation of hydrogen bonds between the hydroxyl groups of titanium aluminate with the aldehyde group of nanocellulose (Ahmad and Guria 2022).

### XRD analysis of nanocomposite membranes

**Fig. 5:**
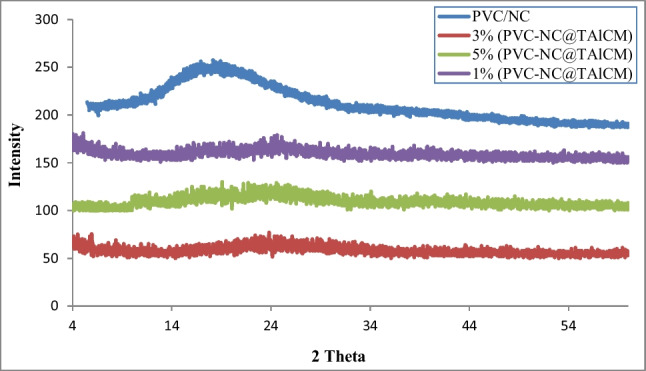
X-ray diffraction scans of PVC/NC and PVC/NC filled with different concentrations of titanium aluminate

XRD pattern of PVC/NC in Fig. 5 shows a diffraction peak at 2θ of 17.3°, while the role of X-ray diffraction of the inorganic nanocomposite, e.g., TiAl_2_O_4_, can be investigated through the entire measurement range. In this regard, the XRD diffraction patterns of PVC-NC@TALCM at 1%, 3%, and 5% of titanium aluminate exhibit the varied type of crystalline phase’s peak; this reveals the presence of some crystalline peak indicating the complete homogenous distribution of TiAl_2_O_4_ in the PVC/NC membrane matrix. Furthermore, the increase in TiAl_2_O_4_% leads to a sharp, intense peak at about 2θ of 24.13, 25.93, and 33.5 for 1%, 3%, and 5% of TiAl_2_O_4_ respectively (Fahoul et al. 2021). Multiple peaks with lower intensities appear at 2ϴ of 27.8, 34.6, 43.59, and 50.35, 38.17, and 48.55, attributed to (220) and (311) of TiAl_2_O_4_%, respectively. The previous interpretation points to the formation of multiple phases in the new material composed of semicrystalline and amorphous phases.

Furthermore, an increase in TiAl_2_O_4_ concentrations increases peak sharpness and hence sample crystallinity, forming a lamellar composite structure. The diffraction peaks that arise are indexing to TiAl_2_O_4_ with a face-centered cubic spinal structure. The TiAl_2_O_4_ spinal phase formed utterly. In addition, there were no impurities found in the prepared sample (Kunde and Sehgal 2020). The Scherer equation estimated the average crystallite size to be around 100 nm.

### SEM analysis of prepared membranes

**Fig. 6:**
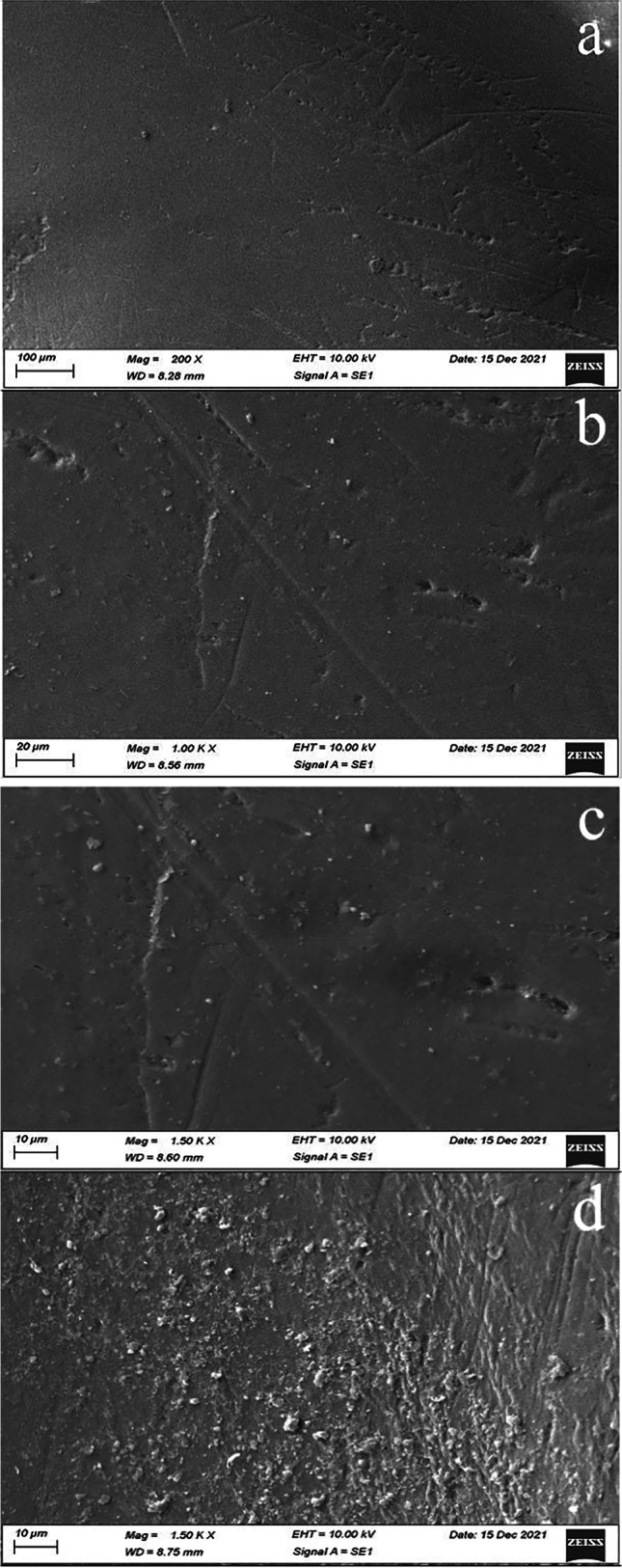
SEM images of blank (**a**), PVC-NC@TALCM 1% (**b**), PVC-NC@TALCM 3% (**c**), and PVC-NC@TALCM 5% (**d**)

SEM image of blank and PVC-NC@TALCM with different ratios (1, 3, and 5%) in Fig. 6a indicates a stable membrane of PVC-NC@TALCM, which was obtained through the preparation process. We note that the surface of the blank membrane is smooth and flat with the fusion of cellulose nanoparticles with the interior of the prepared membrane. Due to physicochemical interactions, the adsorption layer is internal, not external; a stable matrix forms when NC adds to PVC. The matrix becomes strengthened after adding TiAl_2_O_4_ due to the physical reinforcement. The subsequent addition of TiAl_2_O_4_ to the matrix provides sufficient crosslinking, which enhances the overall functional performance and roughness.

SEM images in Fig. 6b reveal the growth of nano/micro clusters of TiAl_2_O_4_ nanotubes through the membrane surface. The clusters are non-uniform in size and non-regular in shape. The tubes are very slick, with an outer diameter of 550 nm and a tube length of 825 ± 51.7 nm. Figure 6c shows irregular clusters almost similar to the morphology of titanium aluminate nanoparticles in Fig. 6b. Increasing the TiAl_2_O_4_ ratio in the membrane increased the size of overgrowing micro clusters despite a drop in their number. Insets with increased TiAl_2_O_4_, as shown in Fig. 6d, where the TiAl_2_O_4_ appeared as uniform circular holes and in a homogenous distribution.

### TGA analysis of prepared membranes

**Fig. 7:**
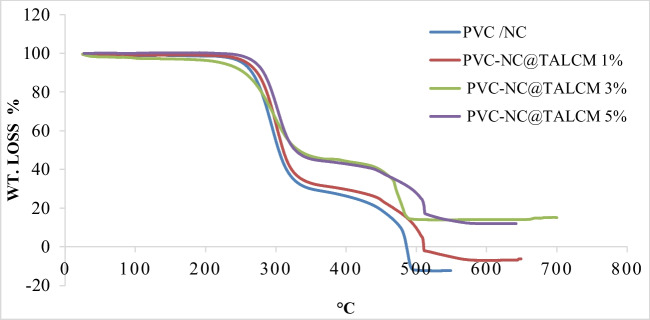
TGA of PVC-NC and PVC-NC@TALCM with different concentration

By analyzing the thermal stability of the prepared membranes, it can be said that the addition of titanium aluminate (TiAl_2_O_4_) helped in the thermal stability of the primary polymer PVC/NC, as shown in Fig. 7. It is noted that the degradation of PVC/NC occurs in three stages. The first stage is the solvent exit, a tiny stage because a small percentage of the solvent in the membrane ends at 150 °C, followed by a second stage that intersects around the exit of functional groups from the base polymer and ends at 334 °C. The final stage is carbonation, which ends at 485 °C and expresses the gaseous vacuum of carbon gas, affecting the analysis result at the end of the slit.

Like other composites that contain titanium aluminate, the increasing proportion of titanium aluminate in the composite has resulted in an increase in the crushing of materials and an expansion in the extent of the crushing stages. It is noted that the first stage is 150 °C, while the second stage is much at 323, 345, and 246 for the ratios from 1–3% for titanium aluminate, which confirms the high thermal stability of the composite for the original membrane. Also, it is noted that the burnt sample decreased with increased titanium aluminate percentage, confirming its thermal stability. Additionally, the intermediate stages appear for the essential components of the nanomaterials used, Al and TiO_2_.

### Performance evaluation of adsorption membranes

#### Effect of initial dye concentrations on the uptake efficiency

**Fig. 8:**
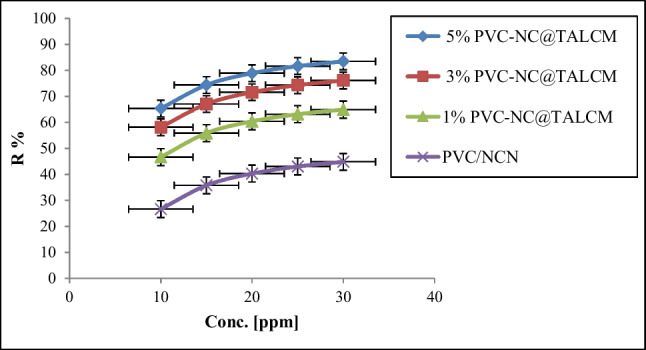
Effect of initial MB concentrations on the adsorption and removal efficiency

The influence of initial dye concentrations on the absorption efficiency of the PVC/NC@TALCM membrane is shown in Fig. 8. With increasing MB concentration, the removal efficiency of PVC/NC@TALCM membrane increased from 65.3 to 83.4%, corresponding to MB concentrations of 10 and 30 ppm in the case of 5% PVC/NC@TAlCM. In contrast, in the case of 3 and 1% membrane ratio, the removal efficiency was 73 and 65.3%, and 41% for PVC/NC, respectively. The adsorption of MB dye on PVC-NC@TALCM membrane is enhanced by increasing the initial concentration of MB dye; this may be due to the complete saturation of the PVC/NC@TAlCM membrane adsorption sites in the case of the 3 and 1% membrane ratio. Whereas, in the case of 5% PVC-NC@TALCM, the increased adsorption of MB dye is most likely due to an increase in the ratio of adsorption sites on the membrane surface compared to the dye concentration, as the driving force generated by increasing the dye concentration increases, and becomes sufficient to overcome the mass transfer between the solid and liquid phases and enhance the adsorption process progress and removal efficiency (Gharbani and Mehrizad 2022).

The adsorption of MB dye on the PVC-NC@TALCM membrane increases with increasing initial concentration due to the availability of more adsorption sites on the membrane surface, resulting in a higher driving force for the adsorption process and an improvement in membrane removal efficiency. However, the relationship between the initial concentration of MB dye and the adsorption capacity of the membrane may not be linear, and there may be a saturation point beyond which further increases in dye concentration do not lead to a corresponding increase in adsorption capacity. Further studies are required to determine the optimal conditions for using this membrane for the effective removal of MB dye from wastewater.

#### Adsorbent dose effect

**Fig. 9:**
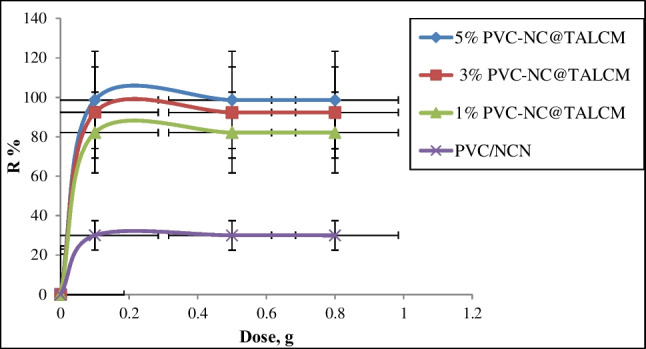
Effect of adsorbent dose on MB adsorption

At all indicated mixing ratios (1, 3, 5%), the maximum removal efficiency of MB dye was (88.2, 97.6, and 98.6%) respectively, as shown in Fig. 9. The presence of different adsorption sites enhanced the available surface area for the adsorption process to complete, leading to a high removal efficiency in the sequence of 5% PVC-NC@TALCM > 3% PVC-NC@TALCM > %1 PVC-NC@TALCM > PVC-NC (Sabarish and Unnikrishnan 2018a, b).

Also, it can say that the TiAl_2_O_4_ composite in the membrane enhances the adsorption process of the dye in cooperation with the cellulose nanoparticle distributed in the polyvinyl chloride supporting membrane layer; this helps to increase the adsorption with an increase in the ratio of the titanium aluminate as shown in Fig. 9.

#### Effect of pH

**Fig. 10:**
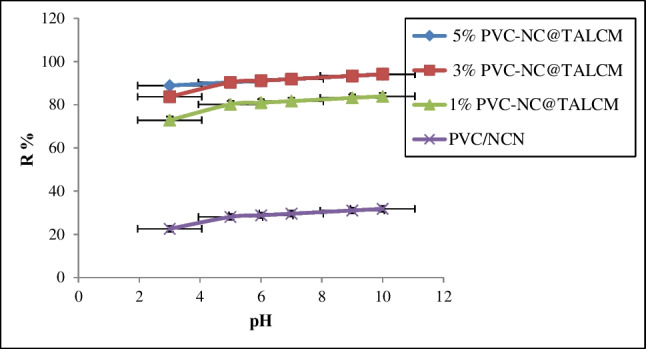
Effect of pH on the adsorption of MB

The influence of pH on MB adsorption by PVC-NC@TAlCM at different ratios (1, 3, and 5%) membranes was investigated, with the findings shown in Fig. 10. The uptake efficiency initially increased with increasing pH for all composite membrane ratios and reached its maximum at pH = 10; these results agree with many researchers [35]. Increasing the pH of the methylene blue dye solution causes electrostatic reactions between the MB dye and the PVC-NC@TAlCM membrane. It is observed mainly in cases of 5% and 3% with a maximum removal efficiency of 98.6%, leading to an increase in the percentage of negative hydroxyl ion, and this leads to an increase in the dye uptake process on the surface of the nanocomposite membrane (Lu et al. 2018). However, the positive charges on the surface of the PVC-NC@TAlCM adsorbent membrane and the positive charge of the methylene blue dye solution compete at lower pH levels.

### Adsorption kinetics

The behavior of uptakes MB by blank and PVC-NC@TALCM nanocomposite adsorption kinetics was investigated using pseudo-first and pseudo-second-order models applied to batch adsorption findings.

**Fig. 11:**
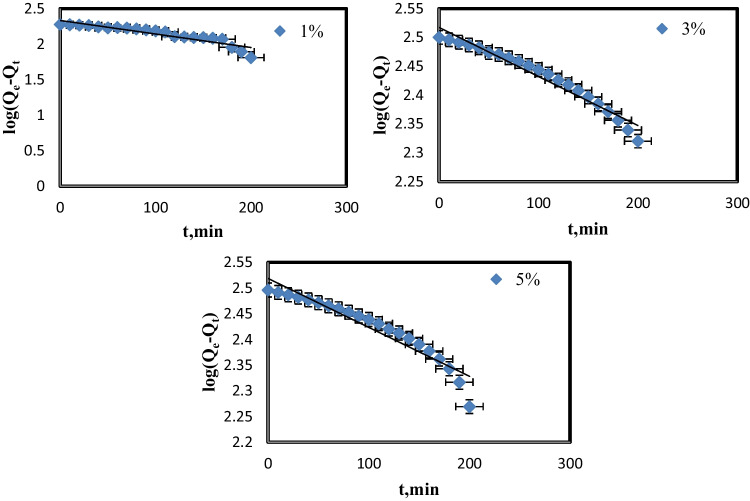
Pseudo-first-order adsorption of MB on nanocomposite membranes (5, 3, 1%)

The pseudo-first-order model’s linear form showing below Eq. 4:

4 $$\mathrm{log}({\mathrm{q}}_{\mathrm{e}}-{\mathrm{q}}_{\mathrm{t}})={\mathrm{logq}}_{\mathrm{e}}-\frac{{\mathrm{k}}_{1}\mathrm{t}}{2.303}$$

where *q*_e (_mg g^−1^) is the adsorption capacity at equilibrium and *q*_t_ (mg g^−1^) is the adsorption capacity at time *t* (min), and *k*_1_ (min^−1^) is the pseudo-first-order rate constant. The slope and intercept of log (*q*_e_-*q*_t_) plots vs. t, as illustrated in Fig. 11, were used to determine the constants *q*_e_, k_1_, and correlation coefficients *r*^2^ (Vedula and Yadav 2022).

The linear expression for the pseudo-second-order rate expression is (Eq. 5):

5 $$\frac{\mathrm{t}}{{\mathrm{q}}_{\mathrm{t}}}=\frac{1}{{\mathrm{k}}_{2}{{\mathrm{q}}_{\mathrm{e}}}^{2}}+\frac{1}{{\mathrm{q}}_{\mathrm{e}}}\mathrm{t}$$

**Fig. 12:**
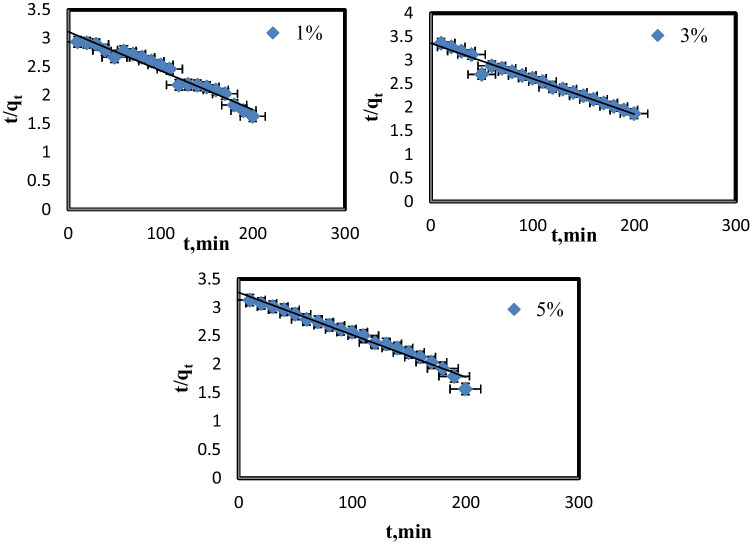
Pseudo-second-order adsorption of MB on nanocomposite membrane (5, 3, 1%)

*Q*_e (_mg g^−1^) is the adsorption capacity at equilibrium, and *q*_t_ (mg g^−1^) is the adsorption capacity at time *t* (min), while *k*_2_ (g mg^−1^ min^−1^) is the rate constant of pseudo-second-order. It can derive the rate constants *k*_2_, *q*_e_, and correlation coefficients *r*^2^ using linear plots of t/qt vs. t. (Fig. 12).

Two kinetic models’ parameters were shown in Table 2, with the correlation coefficient (*r*^2^) in the case of pseudo-first-order kinetic models being 0.79, 0.80, and 0.88 for 1, 3, and 5% of PVC-NC@TALCM, respectively. Whereas, *r*^2^ ranged from 0.99 to 1 in every case when the pseudo-second-order kinetic model was used at 25 °C, indicating that the correlation coefficients (*r*^2^) derived from the pseudo-second-order kinetic model are significantly higher than correlation coefficients derived from the pseudo-first-order kinetic model.

**Table 2:** Calculated kinetic parameters for the adsorption of MB on the membrane

Pseudo-first-order		Pseudo-second-order	
Models	MB	Models	MB
5%			
*k*_1_ (min^−1^)	0.00095	*k*_2_ (× 10^−3^ g mg^−1^ min^−1^)	1.5243
*q*_e_ (Cal) (mg g^−1^)	1.5297	*q*_e_ (Cal) (mg g^−1^)	134.69
*q*_e_ (Exp) (mg g^−1^)	313.57	*q*_e_, (Exp) (mg g^−1^)	141.83
*r*^2^	0.95	*r*^2^	0.99
3%			
*k*_1_ (min^−1^)	0.000849	*k*_2_ (× 10^−3^ g mg^−1^ min^−1^)	1.5062
*q*_e_ (Cal) (mg g^−1^)	2.2517	*q*_e,_ (Cal) (mg g^−1^)	132.801
*q*_e_ (Exp) (mg g^−1^)	316.265	*q*_e_ (Exp) (mg g^−1^)	140.48
*r*^2^	0.85	*r*^2^	0.98
1%			
*k*_1_ (min^−1^)	0.00160	*k*_2_ (× 10^−3^ g mg^−1^ min^−1^)	1.2065
*q*_e_ (Cal) (mg g^−1^)	4.3533	*q*_e_ (Cal) (mg g^−1^)	147.97
*q*_e_ (Exp) (mg g^−1^)	367.58	*q*_e_ (Exp) (mg g^−1^)	163.28
*r*^2^	0.80	*r*^2^	0.98

We may infer that both variables *q*_e_ and *r*^2^ indicated the adsorption of MB onto composite membrane followed the pseudo-second-order kinetic model, indicating that the chemical adsorption process is the rate-limiting step (Hisada et al. 2019).

### Adsorption isotherm

The adsorption isotherm was investigated to clarify MB’s adsorption performance onto PVC-NC@TALCM membranes. Freundlich and Langmuir’s models were applied to discover the appropriate model to explain MB adsorption [39].

*C*_e_ is the equilibrium concentration of adsorbate (mg/l) in the Freundlich and Langmuir model, and *q*_e_ (mg/g) is the adsorbent’s equilibrium adsorption capacity. In contrast, *q*_m_ (mg/g) is the maximum adsorption capacity. Table 3 illustrates the linear form of this model.

Freundlich and Langmuir constants (L/mg) are denoted by *K*_L_ and *K*_f_, respectively. *n* denotes the adsorption intensity, while *K*_f_ denotes the maximal adsorption capacity (mg/g). The slope and intercept of the plot *C*_e_/*q*_e_ vs. *C*_e_ were used to get the Langmuir maximum uptake *q*_m_ and *K*_L_; the Freundlich constants *n* and *K*_f_ were determined by plotting ln *q*_e_ versus l*n C*_e_.

Adsorption favorability was typically predicted using the value of 1/*n*. If 1/*n* = 0, adsorption is irreversible, beneficial if (0 < 1/*n* < 1), and unfavorable if 1/*n* > 1 (Hebbar et al. 2018).

**Fig. 13:**
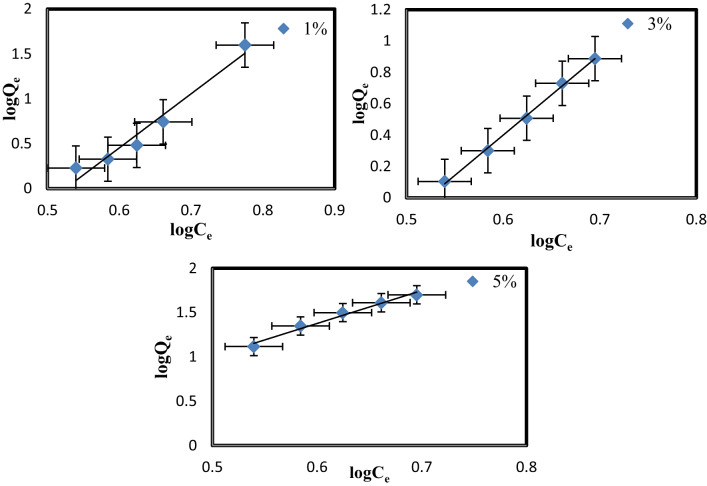
Freundlich adsorption isotherm of MB onto PVC-NC@TAlCM (1, 3, 5%) nanocomposite

**Fig. 14:**
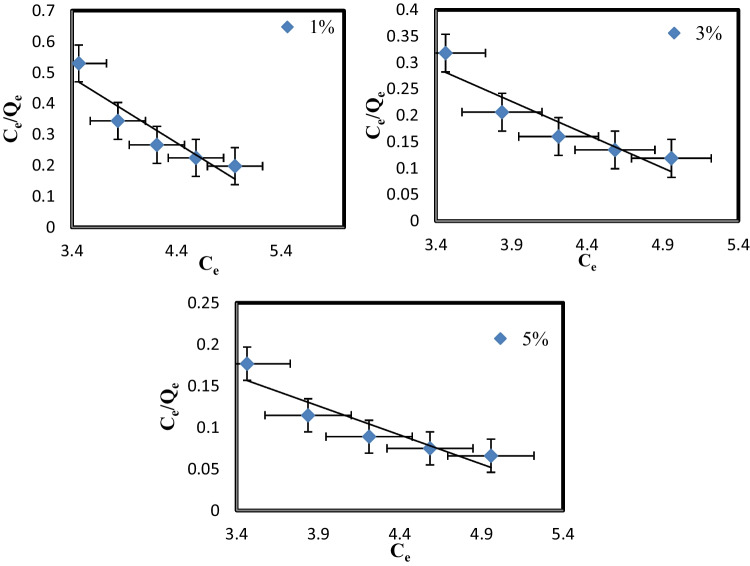
Langmuir adsorption isotherm of MB onto PVC-NC@TAlCM (1, 3, 5%) nanocomposite

Table 3 displays the isotherm constants determined by linearly fitting the experimental data, (Figs. 13, 14). The current experimental findings fit the Freundlich model of adsorption in all cases. This result shows that incorporating TiAl_2_O_4_ into PVC-NC@TALCM alters the membrane surface and enhances an adsorption protocol described by Freundlich.

The Langmuir and Freundlich isotherms were used to characterize the connection between the number of MB adsorbed and its equilibrium concentration in solution at room temperature, the findings are shown in Table 3.

**Table 3:** Langmuir’s and Freundlich calculated isotherms

Isotherm equation	Langmuir	Isotherm constant	Freundlich
5%			
*q*_m_ (mg/g)	14.2974	*K*_f_	2.3302
*K*_L_(L/mg)	35.8582	*n*	0.26
*r* ^2^	0.92	*r* ^2^	0.99
3%			
*q*_m_ (mg/g)	7.94305	*K*_f_	14.55
*K*_L_(L/mg)	11.06735	*n*	0.19
*r* ^2^	0.93	*r* ^2^	0.99
1%			
*q*_m_ (mg/g)	4.7654	*K*_f_	23.5695
*K*_L_(L/mg)	3.9842	*n*	0.16
*r* ^2^	0.92	*r* ^2^	0.985

### Adsorption thermodynamic

**Fig. 15:**
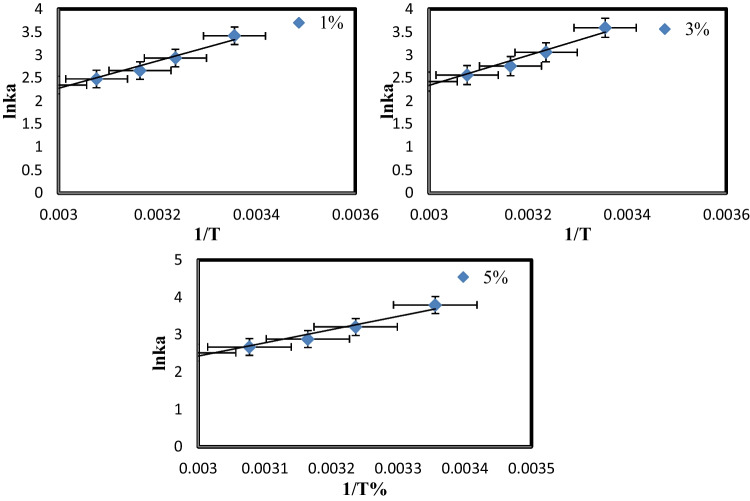
The linear form of vant Hoff equation of PVC-NC@TAlCM (1, 3, 5%) nanocomposite

Thermodynamics studies also elucidate adsorption mechanisms. *K*c, Δ*S*, Δ*G*, and Δ*H* for adsorption were calculated by Vant Hoffer equation (Eq. 6, 7 and 8), as shown in Fig. 15.

6 $${\mathrm{k}}_{\mathrm{c}}=\frac{{\mathrm{q}}_{\mathrm{e}}}{{\mathrm{c}}_{\mathrm{e}}}$$

7 $${\mathrm{lnk}}_{\mathrm{c}}=\left(\frac{\Delta \mathrm{S}}{\mathrm{R}}\right)-\left(\frac{\Delta \mathrm{H}}{\mathrm{R}\times \mathrm{T}}\right)$$

8 $$\Delta \mathrm{G}=-\mathrm{R}\times \mathrm{T}\times {\mathrm{lnK}}_{\mathrm{c}}$$

*T* (K) is the absolute temperature, and *R* (8.314 Jmol^−1^ K^−1^) is the universal gas constant. At the same time, *K*_c_ is the thermodynamic equilibrium constant at different temperatures, Δ*G* is the Gibbs’ free energy in kJ mol^−1^, Δ*H* is the enthalpy change in kJ mol^−1^, and Δ*S* is the entropy change in J mol^−1^ K^−1^ (Gharbani and Mehrizad 2022).

The calculated values for all parameters at MB C0 = 200 mg L^−1^ were indicated in Table 4. For the adsorption system, Δ*G* was negative for all ratios. As a result, physical adsorption occurred spontaneously. The negative Δ*G* value of MB adsorption by membrane surface increased as the temperature rose in all cases, indicating that the MB dye’s adsorption strength also increased. Because of the positive Δ*H* (24,705.2, 26,974.01, 29,391.36 Jmol^−1^) for 1, 3, and 5% of PVC-NC@TAlCM, respectively, MB adsorption is endothermic. The positive Δ*S* (55.19024, 61.45884, 67, 99863 J mol^−1^ K^−1^) for 1, 3, and 5% PVC-NC@TAlCM, respectively, indicated that the membrane surface had high MB affinity (Gohr et al. 2022).

**Table 4:** Thermodynamic parameters of the adsorption process at different temperatures

*T* (^°^C) MB	Δ*G* (J/mol)	Δ*S* (J/mol K)	Δ*H* (J/mol)
5%			
25	− 9389.04	67.99863	29,391.36
36	− 8233.35		
43	− 7568.07		
52	− 7208.97		
61	− 6985.23		
3%			
25	− 8892.87	61.45884	26,974.01
36	− 7854.77		
43	− 7249.98		
52	− 6924.31		
61	− 6721.75		
1%			
25	− 8462.78	55.19024	24,705.18
36	− 7543.49		
43	− 6994.95		
52	− 6699.45		
61	− 6515.63		

### Self-cleaning properties of prepared membrane based on contact angle measurement

It is essential to know that the contact angle measurement only indicates the self-cleaning behavior of the membrane surface. Based on the contact angle measurements, the PVC/nanocellulose@TiAl_2_O_4_ membrane exhibits a low contact angle, indicating that the surface is hydrophilic and may exhibit self-cleaning behavior. Our measurements yielded an average contact angle of 43°, which is lower than the control sample (PVC/NC) which recorded 89.5°, and by comparing our results with the previous studies on hydrophilic membranes, it is found that the measured contact angles of casting PVC/nanocellulose@TiAl_2_O_4_ membrane films are in line with recent literature values (Hurwitz et al. 2010). It can be said that the PVC/nanocellulose@TiAl_2_O_4_ membrane exhibits a self-cleaning property based on the low contact angle (43°) measurement. This property is likely due to the combined effect of the TiAl_2_O_4_ nanoparticles and the nano cellulose fibers on the surface of the membrane.

Additionally, the results can suggest that the PVC/nanocellulose@TiAl_2_O_4_ membrane surface has high surface energy, which can prevent the adhesion and accumulation of MB dye. This is due to the hydrophilicity of the TiAl_2_O_4_ nanoparticles and the high roughness of combined nano cellulose fibers, which create a hierarchical roughness on the surface of the membrane. This roughness can trap air pockets, preventing contact with the water droplets and promoting the self-cleaning effect in the case of MB dye filtration (Hui Ting et al. 2023).

### Adsorption process mechanism

**Fig. 16:**
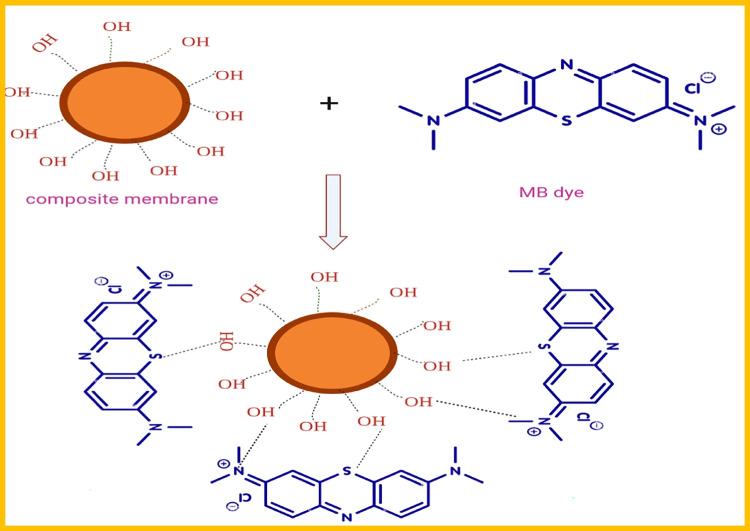
Zeta potential curve as a function of solution pH obtained for (PVC/NC@TAlCM)

The possible adsorption mechanism of MB on the surface of (PVC/NC@TAlCM) was illustrated in Fig. 16. According to the existing functional groups on the (PVC/NC@TAlCM) surface, the MB adsorption mechanism can be assigned to the various interactions such as electrostatic, hydrogen bonding interactions, van der Waals, and π-π stacking. Therefore, the zeta potential was examined to understand better the contact forces between the membrane surface and the MB. The zeta potential of the modified membrane surface was 4.8–1.2 mV at pH 3 and 10, suggesting that the electrostatic interaction was the most important contact between the composite membrane surface and the MB. That means, the adsorption of MB from aqueous solution by (PVC/NC@TAlCM) is strongly dependent on the interaction of the polar functional groups (− SO_3_) of MB molecule with the OH group of the composite membrane surface (PVC/NC@TAlCM). Similar observations have been reported for the adsorption of MB on other surfaces (Wang et al. 2022a, b, c).

### The novelty of the prepared nanocomposite (PVC-NC@TiAl_2_O_4_)

To increase the water treatment operating efficiency process and energy savings, the nanofiltration membranes were prepared for semi-industrial purposes. Therefore, a new nanofiltration membranes (PVC/NC) with excellent initial separation performance for industrial wastewater treatment were synthesized and integrated with nano-sized TiAL_2_O_4_ to be more flexible, durable, and high biological and chemical resistance. Additionally, the self-cleaning is also possible, enhancing chemical properties and reducing the number of chemicals used.

For this purpose, polyvinyl chloride-nanocellulose (PVC/NC) membranes are prepared by the phase inversion method (by dissolving and blending PVC and NC solution). In addition, varying amounts of nano-sized TiAl_2_O_4_ are employed as an adsorbent to generate nanocomposite membranes with high removal efficiency. The impact of the polymer binder blend ratio (PVC to NC) and TiAl_2_O_4_ concentration on the physicochemical properties of membranes is also investigated.

Based on Table 5, it appears that the nanofiltration membranes (PVC-NC@ TiAl_2_O_4_) have a high rejection rate of 98.6% for MB dyes with an initial concentration of 150 mg/L. This is a significant result, as it indicates that the nanofiltration membranes are effective at removing the dyes from the solution.

**Table 5:** Various adsorption-membrane systems for various pollutants removal

Type of membranes	Type of adsorbents	*C*_0_, mg/l	*R*%	Pollutants	Ref
UF	(PVA/PDADMAC/ZSM-5)	20	95%	Methyl orange dye	(Sabarish and Unnikrishnan 2018a, b)
NF	CuTz-1/graphene oxide (CuTz-1/GO)	50	94.9%	Methylene blue dye	(Zhou et al. 2021)
MF	Powdered activated carbon	50	90%	Synthetic organic contaminants	(Li 2014)
UF	(PVA/CMC/ZSM-5 zeolite)	100	97%	Methylene blue dye	(Sabarish and Unnikrishnan 2018a, b)
UF	Bentonite	50	97%	Methylene blue dye	(Al-Bastaki and Banat 2004)
NF	(PVC-NC@TALCM)	150	98.6%	Present study	

As illustrated in Table 5, when comparing the result of PVC-NC@ TiAl_2_O_4_ membranes with other adsorbents and membrane types, it appears that the PVC-NC@ TiAl_2_O_4_ membranes are among the most effective at removing dyes. In fact, their rejection rate is higher than that of most of the other adsorbents and membranes listed.

It is important to note that the effectiveness of a particular adsorbent or membrane will depend on the specific pollutant being targeted, as well as other factors such as the initial concentration of the pollutant and the conditions under which the adsorption or filtration is taking place. However, based on the information in Table 5, it appears that the PVC-NC@ TiAl_2_O_4_ membranes are a promising choice for dye removal. Overall, the high rejection rate of the PVC-NC@ TiAl_2_O_4_ membranes suggests that they are a promising technology for dye removal. However, further research is needed to determine their effectiveness under different conditions and for different types of dyes.

## Conclusion

Novel PVC-NC@TALCM nanocomposite filtration membranes were successfully prepared by adding TiAl_2_O_4_, effectively modifying the PVC-NC@TALCM matrix according to XRD, FTIR, SEM, and TGA for the removal of methyl blue from industrial wastewater. The efficiency of 5, 3, and 1% PVC/NC@TALCM nanocomposite membrane was 98.6, 92.29, and 82.09%, respectively, at pH 10 and 30 ppm (initial dye concentration). The Freundlich model can describe the adsorption isotherm of MB on PVC-NC@TALCM nanocomposite membrane, and the adsorption kinetics followed the pseudo-second-order model for all ratios. Maximum adsorption capacity (*q*_e_) was found to be 134.69 mg g^−1^. The adsorption process was found to be spontaneous and endothermic, accompanied by an increase in entropy. It was concluded that the PVC-NC@TALCM nanocomposite membrane was an economical, environmentally friendly adsorbent for removing MB from industrial wastewater. In addition, it is self-cleaning, which helps in sustainability and will enhance the chemical properties and capabilities of the nanocomposite membranes by reducing the number of chemicals.

## Data Availability

The datasets used and analyzed during the current study are available from the corresponding author upon reasonable request.

## Abbreviations

PVC-NC@TALCM: Polyvinyl chloride nanocellulose@titanium aluminate
PVC: Polyvinyl chloride
NC: Nanocellulose
Ti_2_ALO_4_: Titanium aluminate
THF: Tetrahydrofuran
DMF: Dimethylformamide
